# Temperature and feeding frequency: interactions with growth, immune response, and water quality in juvenile Nile tilapia

**DOI:** 10.1186/s12917-024-04366-4

**Published:** 2024-11-18

**Authors:** Sara Hamed, Seham El-Kassas, Haitham G. Abo-Al-Ela, Safaa E. Abdo, Usama A. Abou-Ismail, Radi A. Mohamed

**Affiliations:** 1 Department of Aquaculture, Faculty of Aquatic and Fisheries Sciences, Kafrelsheikh University, Kafrelsheikh, Egypt; 2 Animal, Poultry and Fish Breeding and Production, Department of Animal Wealth Development, Faculty of Veterinary Medicine, Kafrelsheikh University, Kafrelsheikh, 33516 Egypt; 3 Genetics and Biotechnology, Department of Aquaculture, Faculty of Fish Resources, Suez University, Suez, 43221 Egypt; 4 Genetics and Genetic Engineering, Department of Animal Wealth Development, Faculty of Veterinary Medicine, Kafrelsheikh University, Kafrelsheikh, 33516 Egypt; 5 Department of Husbandry and Development of Animal Wealth, Faculty of Veterinary Medicine, Mansoura University, Gomhoria St., Mansoura, 35516 Egypt

**Keywords:** Antioxidant responses, Daily meal intake, Growth performance, Innate immunity, Nile tilapia, Water temperature

## Abstract

**Background:**

Water temperature and feeding frequency are critical abiotic factors regulating the growth and immune function of aquatic organisms. This study investigated the effects of water temperature and feeding frequency on growth and immune function in Nile tilapia (*Oreochromis niloticus*) over two months. A total of 360 juvenile fish (average weight: 20.00 ± 1.26 g) were divided into six groups, each with three replicates, based on a combination of three water temperatures (26, 28, and 30 °C) and two feeding frequencies (either 1 or 2 meals per day).

**Results:**

At 30 ºC and 28 ºC, water electrical conductivity and total dissolved salts increased, while total ammonia nitrogen and dissolved oxygen rose slightly in groups fed twice daily, with a significant interaction between temperature and feeding frequency. The group at 30 ºC with two meals per day showed the highest final body weight (FBW). The interaction between temperature and feeding frequency significantly influenced FBW, total feed intake, and body thickness. Fish at 30 ºC exhibited upregulated hepatic growth hormone receptor 1 and insulin-like growth factor 1, while those at 28 ºC with one meal per day, as well as those at 30 ºC regardless of meal frequency, also showed increased expression of hepatic fatty acid binding protein and intestinal cluster of differentiation 36. Fish at 30 ºC had upregulated leptin levels and downregulated cholecystokinin, while those at 26 ºC displayed the opposite trend, particularly with one meal daily. Higher temperatures significantly boosted serum IgM, superoxide dismutase (SOD), and lysozyme (LYZ) levels, with meal frequency also affecting malondialdehyde, IgM, and SOD levels. Additionally, 30 ºC enhanced the hepatic expression of mucin-like protein (*muc*), oligo-peptide transporter 1 (*pept1*), interleukin 1, *nf-κB*, complement C3, *lyz*, *sod*, catalase, and glutathione peroxidase, with twice-daily meals having a more pronounced effect. Conversely, 28 ºC with one meal per day upregulated some of these genes, such as *muc*, *pept1*, and *sod*.

**Conclusions:**

Overall, 30 ºC with two meals per day significantly improved the growth and health of juvenile Nile tilapia, while 28 ºC with two meals maintained satisfactory performance.

## Introduction

In Egypt and many other countries, Nile tilapia (*Oreochromis niloticus*) stands out as a valuable fish species due to its adaptability to various environmental challenges and its high market value [1]. According to the FAO [2], Nile tilapia is globally recognized as the 3rd most farmed fish species, with a production of 6.7 million tons recorded in 2023 [3]. However, several challenges impact Nile tilapia productivity, including culturing density, feeding frequency, and water quality parameters such as temperature, dissolved oxygen (DO) concentration, pH, and ammonia concentration [1]. Therefore, it is crucial to investigate how alterations in these factors influence the performance of Nile tilapia.

Water temperature is identified as a crucial abiotic factor regulating fish growth and health at all stages of development [4, 5]. Global climate changes significantly affect temperature patterns, consequently impacting water temperature. Extremes in pond temperature can alter fish growth, as well as hemato-physiological, metabolic, immune, and molecular responses [4, 6]. Higher water temperatures, within optimal levels, increase food demand, energy requirements, and fish metabolic rates [6, 7]. In this context, the growth of most fish species, including tilapias, increases with rising water temperature to a certain point, but then declines abruptly when temperatures exceed physiological tolerance [1]. Conversely, a reduction in water temperature lowers fish growth [1]. Therefore, determining the optimum temperature range for maximum growth and physiological performance is crucial.

The feeding composition and frequency (FF) are critical modulatory factors for fish growth, including in tilapias, as they affect food conversion and weight gain [8, 9]. Increasing the FF reduces aggressive feeding behavior, ensuring faster fish growth and uniformity in harvest size by facilitating feed pickup, thereby improving fish growth performance, survival, body composition [10], and water quality [11]. Moreover, FF has been found to modulate digestive enzyme activities, specifically trypsin in both pancreatic and intestinal segments in large yellow croaker larvae. Increasing FF also resulted in elevated body levels of crude protein and crude lipid without significant differences in fatty acid composition [12].

Considering both factors, water temperature and FF, Wang et al. [13] found that, among the temperatures studied (20, 24, and 28 °C), the fish exhibited the highest growth performance at 28 °C. Furthermore, increasing the FF to six times per day recorded the highest performance. FF also significantly affected protein and lipid retention efficiencies, while temperature significantly influenced body protein content [13]. Temperature has been shown to modulate lipid, carbohydrate and protein metabolism in Nile tilapia, affecting digestible energy [14]. Similarly, FF had a significant impact on lipid metabolism-related genes. For instance, during fasting, hepatic lipoprotein lipase regulates serum triacylglycerol levels, while fatty acid synthetase expression is influenced by nutrient availability [15].

Water temperature and FF were found to impact reproductive performance, with high temperatures and low FF reducing the breeding rate of coral reef damselfish (*Acanthochromis polyacanthus*) [16]. In zebrafish, increased feeding frequencies led to greater body length and weight, while moderate FF (once or three times per day) resulted in more successful breeding, as reflected by higher mean fecundity and embryo viability [17].

Climate changes affect global temperatures, including water temperature, altering feed availability and thus frequencies, leading to increased disease incidence [4, 18]. Proposed increases in climate temperatures are expected to influence endemic diseases in water environments, increasing the prevalence of diseases such as proliferative kidney disease and white spot, as well as outbreaks of koi herpesvirus [18]. Fish diseases are often associated with water temperature. An increase in water temperature creates favorable conditions for pathogens to thrive and develop diseases [18]. Additionally, FF has been shown to modulate the health status of fish [19, 20]. Both low FF and high FF disrupt the immune function of fish. Low FF results in a declined immune response and disease resistance; however, high FF leads to oxidative stress and, therefore, immunosuppression [19].

In a prior study, researchers found that water temperature, FF, and the percentage of dietary protein significantly influenced the growth, behavior, and water quality of Nile tilapia [21]. Building on this, our study hypothesizes that variations in water temperature and FF, as well as their interactions, will influence the overall performance of Nile tilapia. The aim is to explore these effects more comprehensively by examining a broader range of parameters. Specifically, we will assess the impact of different water temperatures and feeding frequencies on growth performance, immune and antioxidant responses, blood biochemical composition, and gene expression related to lipid metabolism and immunity. Additionally, the study investigates how reducing feeding frequency to once or twice per day influences the fish’s overall performance.

## Materials and methods

### Fish source and rearing management

The present study involved 360 Nile tilapias, *Oreochromis niloticus*, with an average body weight of 20.00 ± 1.26 g. The fish were procured from a commercial hatchery in Kafrelsheikh Governorate, Egypt, and transported in plastic tanks equipped with air pumps to the laboratory. There, they underwent a two-week acclimatization period, during which they were fed a commercial tilapia diet (ALEKHWA^®^, Feed Business, Kafrelsheikh, Egypt). The diet composition included 25% crude protein, 5.9% crude fiber, 0.39% available phosphorus, 1.1% calcium, and 2700 kcal/kg metabolizable energy.

**Fig. 1:**
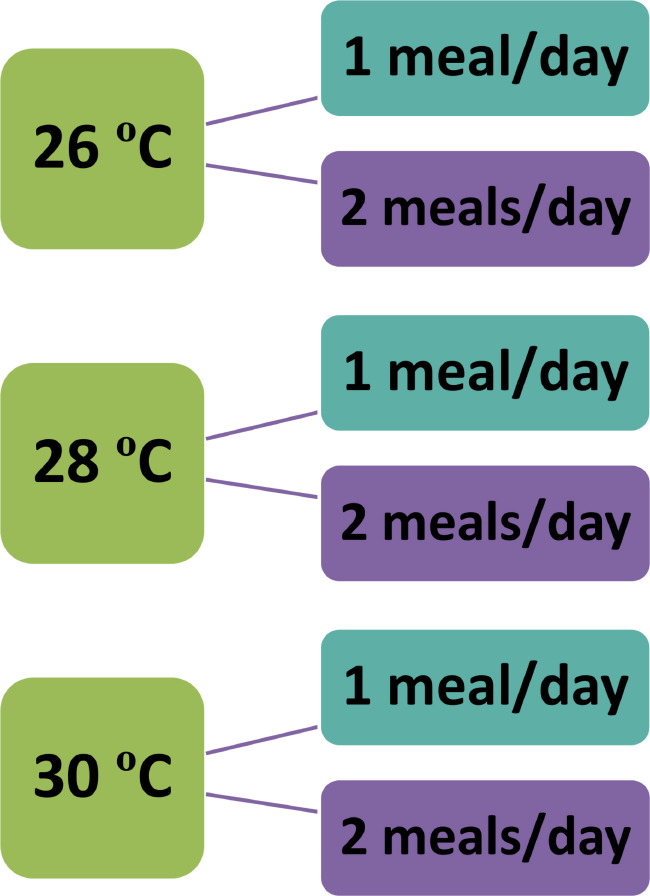
The experimental design

Following acclimatization, the fish were randomly assigned to a 3 × 2 experimental design (Fig. 1). This design included three distinct water temperatures (26, 28, and 30 °C), which represent the biologically relevant range for optimal Nile tilapia growth [22]. To ensure the target temperature was reached and maintained, we used an aquarium heater with a built-in thermostat (SEAJOEWE adjustable 100 W aquarium heater, super short submersible fish tank heater with LED digital display thermostat). Additionally, two feeding frequencies (1 or 2 meals per day) were tested for each temperature, aligning with common aquaculture practices aimed at maximizing performance [23]. This setup resulted in six distinct treatments, each with three replicates (20 fish per tank), for a total of 18 experimental units in the study.

The fish were housed in glass aquaria measuring 80 × 40 × 45 cm³, equipped with an air stone for oxygen supply and a motorized filter (Shark SH-1000 Multi-Function Filter, China). Filters were cleaned every two days to remove waste, and 25% of the water in each aquarium was replaced weekly with clean, dechlorinated water of the same temperature as the experimental conditions. The fish were exposed to a 12/12-hour light-dark cycle per day. The experiment spanned two months, during which the fish were fed a commercial tilapia diet (ALEKHWA^®^, Feed Business, Kafrelsheikh, Egypt).

The feeding rate started at 4% of body mass and was adjusted every two weeks based on the biomass of each aquarium. Throughout the two-month feeding trial, fish in different treatments were fed either once or twice a day at equal intervals.

Water quality assessments were conducted weekly at a fixed time (8 a.m.) in the center of each aquarium using a multi-parameter probe apparatus (HI9829-03042-HANNA^®^ instrument). Parameters measured included DO, water pH, water’s electrical conductivity, and total dissolved solids (TDS). Additionally, total ammonia nitrogen (TAN) was measured using a portable colorimeter (Martini MI 405), and unionized ammonia (UIA) was calculated based on the method described by Zhang et al. [24]. Briefly, the UIA concentration was calculated using TAN, water pH, and water temperature based on the following equation:

$$\:UIA\:(\text{m}\text{g}/\text{L})=\frac{TAN(\text{m}\text{g}/\text{L})}{1+{10}^{(pK-pH)}\:}$$

where:

- **pH** = negative logarithm of the hydrogen ion activity (− log[*H*+]);
- **pK** is the acid dissociation constant for water, calculated as:

$$\:pK=0.09018+\frac{2729.92}{(273.2+T)}$$

where **T** is the water temperature in ºC, which is derived from air temperature.

### Measurement and sampling

#### Growth performance

The initial body weight (IBW) and daily feed intake were recorded to the nearest gram. To calculate feed intake (FI), uneaten feed from each aquarium was collected 20 min after feeding, dried, and weighed. Daily feed intake was determined by subtracting the weight of uneaten feed from the offered amount. At the conclusion of the experiment, the fish were collected using a specific net, dried with filter paper to remove excess water, and individually weighed to record the final body weight (FBW) using a digital balance (PW Balance, ADAM Equipment Co., USA). Body weight gain (BWG) was calculated by subtracting IBW from FBW (BWG = FBW – IBW).

The feed conversion ratio (FCR) was calculated using the formula $\:FCR=\frac{\text{F}\text{e}\text{e}\text{d}\:\text{i}\text{n}\text{t}\text{a}\text{k}\text{e}\:\left(\text{g}\right)}{\text{W}\text{e}\text{i}\text{g}\text{h}\text{t}\:\text{g}\text{a}\text{i}\text{n}\:\left(\text{g}\right)}$. Additionally, the specific growth rate (SGR) % was calculated using the formula SGR% = (ln (FBW in grams) – ln (IBW in grams) x 100) / t (in days). Body length, width, and thickness were individually measured using a measuring board and caliper, following the method described by Bagenal [25].

#### Sampling

At the end of the experimental period, three fish per aquarium (*n* = 9/treatment) were randomly selected and sedated with tricaine methanesulfonate (MS-222) at 35 mg/L for blood and tissue sampling [26, 27]. Blood samples were collected from the tail vein using a sterile syringe. The samples were then left to clot for 30 min before undergoing serum separation through centrifugation at 1,500 x g for 10 min at 4 °C. Subsequently, the serum samples were preserved at − 20 °C for biochemical analysis.

Following the blood sampling, the selected fish were euthanized with MS-222 at 300 mg/L [26, 27] and dissected for liver tissue extraction. The obtained liver tissue was placed in a clean sterile micro-centrifuge tube and rapidly frozen in liquid nitrogen. The frozen samples were then stored at − 80 °C for subsequent RNA extraction.

### Serum biochemical profiling

Serum levels of glucose, total cholesterol, triglycerides (TG), high-density lipoprotein (HDL), and low-density lipoprotein (LDL), as well as total protein and albumin, were measured using specific commercial kits (Biodiagnostic Co., Egypt). The determination of serum globulin levels was made based on the levels of total protein and albumin, utilizing the following equation: globulin = total protein – albumin.

Additionally, liver function enzymes, such as aspartate aminotransferase (AST) in U/L and alanine aminotransferase (ALT) in U/L, along with levels of malondialdehyde (MDA), immunoglobulin-M (IgM), catalase (CAT), sodium oxide dismutase (SOD), and lysozyme (LYZ), were assessed using specific commercial kits (Biodiagnostic Co., Egypt).

### Real-time PCR analysis of selected genes related to growth, feed intake, lipid metabolism, antioxidant activity, and innate immunity

Liver specimens (*n* = 9/treatment) were utilized for RNA extraction using TRIzol (TRIzol™, Invitrogen) in accordance with the manufacturer’s guidelines. The integrity and purity of the extracted RNA samples were assessed through 2% agarose gel electrophoresis and Nanodrop, respectively. Constant concentrations of RNA (2 µg) were employed for reverse transcription and cDNA synthesis using specific cDNA kits (iNtRON Biotechnology). Real-time PCR was then performed for growth-related genes (insulin-like growth factor 1 (*igf1*) and growth hormone receptor 1 (*ghr1*)), feed intake-related genes (cholecystokinin (*cck*) and leptin), lipid metabolism-related genes (fatty acid binding protein (*fabp*) and intestinal cluster of differentiation 36 (*cd36*)), antioxidant-related genes (superoxide dismutase (*sod*), catalase (*cat*), and glutathione peroxidase (*gpx*) and innate immunity-related genes (mucin-like protein (*muc*), oligo-peptide transporter 1 (*pept1*), interleukin 1 (*ilβ1*), interleukin 8 (*il8*), nuclear factor kappa B subunit (*nf-κB*), complement C3 (*c3*) and lysozyme (*lyz*)) using specific primers. The *β*-actin housekeeping gene served as an internal standard (Table 1).

**Table 1:** The sequences of primers used in the study

Gene	Primer	GenBank accession NO	Reference
*β-actin*	F: CAGCAAGCAGGAGTACGATGAG R: TGTGTGGTGTGTGGTTGTTTTG	XM_003455949.2	Abo-Al-Ela et al. [75] and El-Kassas et al. [76]
*ghr1*	F: CAGACTTCTACGCTCAGGTC R: CTGGATTCTGAGTTGCTGTC	MW509678.1	
*igf1*	F: GTTTGTCTGTGGAGAGCGAGG R: GAAGCAGCACTCGTCCACG	NM_001279503.1	
*fabp*	F: CAAGCCCACCACCATCATCT R: TTCCCGTCCTCTATCGTGACA	XM_003444047.5	
*cd36*	F: CCCAAAGCGAACGTCACATT R: ATGTGATGCTGGAGGAAGCAA	XM_003452029.5	
*cck*	F: CAGAAACTCCACGGCAAACA R: TCATACTCCTCTGCACTGCG	NM_001279730.1	
*leptin*	F: AGGCTGGACAAAGACGTACA R: AACCGTTCAAGACCGTCTCT	NM_001301050.1	
*muc*	F: TGCCCAGGAGGTAGATATGC R: TACAGCATGAGCAGGAATGC	XM_025902524.1 XM_025908463.1 XM_025908461.1 XM_019355917.1 XM_025904617.1	Aanyu et al. [77]
*pept1*	F: CAAAGCACTGGTGAAGGTCC R: CACTGCGTCAAACATGGTGA	XM_013271589.3	
*il8*	F: CTGTGAAGGCATGGGTGTGGAG R: TCGCAGTGGGAGTTGGGAAGAA	NM_001279704.1	Abdo et al. [78]
*ilβ1*	F: TCAGTTCACCAGCAGGGATG R: GACAGATAGAGGTTTGTGCC	OR432591.1	
*nf-κB*	F: GAACATCAGACCGACGACCA R: TCTCCGCCAGTTTCTTCCA	XM_003457469.5	
*c3*	F: GGTGTGGATGCACCTGAGAA R: GGGAAATCGGTACTTGGCCT	XM_013274267.3	Esam et al. [79]
*lyz*	F: AAGGGAAGCAGCAGCAGTTGTG R: CGTCCATGCCGTTAGCCTTGAG	XM_003460550.2	
*cat*	F: CCCAGCTCTTCATCCAGAAAC R: GCCTCCGCATTGTACTTCTT	JF801726.1	Abdo et al. [80]
*gpx*	F: CCAAGAGAACTGCAAGAACGA R: CAGGACACGTCATTCCTACAC	DQ355022.1	El-Kassas et al. [81]
*sod*	F: CATGCTTTTGGAGACAACAC R: ACCTTCTCGTGGATCACCAT	XM_003446807.5	El-Haroun et al. [82]

Insulin-like growth factor 1 (*igf1*), growth hormone receptor 1 (*ghr1*), cholecystokinin (*cck*), mucin-like protein (*muc*), oligo-peptide transporter 1 (*pept1*), fatty acid binding protein (*fabp*), intestinal cluster of differentiation 36 (*cd36*), superoxide dismutase (*sod*), catalase (*cat*), glutathione peroxidase (*gpx*), interleukin β 1 (*ilβ1*), interleukin 8 (*il8*), nuclear factor kappa B subunit (*nf-κB*), complement C3 (*c3*), and lysozyme (*lyz*)

The real-time PCR reaction mixes were prepared in duplicates using SensiFast™ SYBR Lo-Rox master mix (Bioline, United Kingdom), with 0.5 µM from the forward and reverse primers, and 2 µL of the cDNA. The reactions were then conducted in the StrateGene MX300P real-time PCR system (Agilent Technologies) with the following thermal cycling conditions: initial denaturation at 95 °C for 15 min, followed by 40 cycles at 95 °C for 15 s, and annealing for 1 min at gene-specific annealing temperatures.

The specificity of amplified PCR products was verified by ensuring the presence of only one peak at the designated melting temperature for each gene in the dissociation curve. The relative expression of the investigated genes was determined using the 2^−ΔΔct^ method, with fish cultured at a water temperature of 28 ºC and fed twice daily serving as the control.

### Statistical analysis

The acquired data were analyzed using the GLM procedure in IBM SPSS Statistics for Windows (SPSS version 22, SPSS Inc., IL, USA). Shapiro–Wilk and Levene tests were employed to verify the normality and homogeneity of variance among the variables in the obtained data, respectively. Two-way ANOVA was then utilized to examine the effects of water temperature, feeding frequency, and their interactions on various parameters. Statistical significance was assessed through Tukey’s HSD test for multiple comparisons. Partial Eta-squared (η²) was calculated to assess the effect sizes of the interactions in our analysis. Results were deemed significant at *P* < 0.05. The presented findings are expressed as means ± SEM. Figure creation was performed using GraphPad Prism 9 software (GraphPrism Software, La Jolla, California, USA).

## Results

### Water quality parameters

The electrical conductivity and total dissolved salts (TDS) of the water in groups raised under 30 ºC exhibited significant differences, with increases of 89 µS/L and 46 mg/L, respectively, compared to those raised under 26 ºC (Table 2). However, compared to the fish raised under 28 ºC, the increases in electrical conductivity and TDS were not deemed significant. A similar trend was observed in total ammonia nitrogen (TAN), which recorded 0.18 ± 0.034 mg/L in the 26 ºC group and 0.10 ± 0.020 mg/L in the 30 ºC group. Water pH and DO remained unchanged among the different groups.

**Table 2:** Water quality parameters of the different groups raised under varied temperatures and feeding frequencies

	Water electric conductivity (µS/L)	TDS (mg/L)	Water pH	TAN (mg/L)	DO (mg/L)
26 ºC					
One M/d	676.86 ± 19.09 ^b^	334.86 ± 9.44 ^b^	7.43 ± 0.095	0.073 ± 0.039 ^c^	3.97 ± 0.257 ^b^
Two M/d	629.14 ± 13.83 ^b^	314.71 ± 6.98 ^b^	7.52 ± 0.131	0.279 ± 0.005 ^a^	5.28 ± 0.125 ^a^
28 ºC					
One M/d	725.71 ± 28.29 ^a^	362.71 ± 14.16 ^a^	7.41 ± 0.096	0.223 ± 0.005 ^a^	3.497 ± 0.483 ^b^
Two M/d	686.00 ± 21.40 ^a^	343.29 ± 10.83 ^a^	7.48 ± 0.099	0.018 ± 0.014 ^c^	4.706 ± 0.327 ^a^
30 ºC					
One M/d	726.86 ± 20.87 ^a^	363.43 ± 10.30 ^a^	7.53 ± 0.102	0.030 ± 0.005 ^c^	4.636 ± 0.098 ^b^
Two M/d	757.43 ± 25.13 ^a^	378.29 ± 12.44 ^a^	7.33 ± 0.106	0.171 ± 0.005 ^a^	4.813 ± 0.240 ^a^
*P* values					
Temperature	0.001	0.001	0.902	< 0.001	0.062
Number of meals	0.297	0.362	0.926	0.002	< 0.001
Interaction*	0.160	0.201	0.332	< 0.001	0.087
Partial Eta squared (η²)**	0.822	0.746	0.135	0.079	0.07

*Donates the water temperature × number of meals interaction. **donates the partial Eta Squared (η²) for the temperature-number of meals interaction. M/d, meals/day; TDS, total dissolved salts; TAN, total ammonia nitrogen; DO, dissolved oxygen. The raw data were obtained from Hamed et al. [21]. Different letters donate statistical significance at *P* < 0.05

In terms of the impact of FF on water parameters, electrical conductivity, TDS, and pH showed slight nonsignificant changes across groups (Table 2). However, TAN and DO experienced slight but significant increases in the group receiving two meals per day. Notably, the TAN parameter revealed a significant interaction between temperature and the number of meals.

### Growth performance

The total feed intake (TFI) reached its highest significant value in the group raised under 28 ºC, receiving one meal per day, compared to the other groups, except for those in the group raised under 30 ºC, which were nonsignificant (Table 3). The group raised under 30 ºC, with two meals per day, exhibited the most significant record among the groups in terms of FBW. Similarly, final body thickness (FT) followed the same trend, although this increase was not significant compared to that recorded in the fish raised under 26 ºC and receiving one meal per day. Changes in total weight gain (TWG), FCR, SGR, final body length (FL), final body width (FW), and condition factor (CF) did not show significant differences among the groups. The interaction between temperature and FF proved to be significant in relation to FBW, TFI, and FT.

**Table 3:** Growth performance of the different groups raised under varied temperatures and feeding frequencies

	IBW (g)	FBW (g)	TFI (g)	TWG (g)	FCR	SGR (%)	FL (cm)	FW (cm)	FT (mm)	CF (%)
26 ºC										
One M/d	21.50 ± 0.30	54.20 ± 2.97 ^b^	57.61 ± 2.67 ^b^	32.7 ± 3.18	1.03 ± 0.052	5.95 ± 0.531	15.28 ± 0.319	4.73 ± 0.110	23.63 ± 0.689 ^ab^	1.63 ± 0.094
Two M/d	21.70 ± 0.24	51.86 ± 2.34 ^b^	56.40 ± 3.160 ^b^	30.16 ± 2.48	1.17 ± 0.065	4.67 ± 0.456	14.13 ± 0.432	4.715 ± 0.232	20.82 ± 0.514 ^b^	1.82 ± 0.240
28 ºC										
One M/d	23.50 ± 0.20	53.39 ± 3.90 ^b^	62.40 ± 3.760 ^a^	29.89 ± 3.92	1.31 ± 0.044	4.94 ± 0.695	14.48 ± 0.488	4.42 ± 0.182	22.86 ± 0.546 ^b^	1.77 ± 0.241
Two M/d	21.00 ± 0.27	52.74 ± 2.70 ^b^	57.40 ± 1.160 ^b^	31.74 ± 2.74	1.11 ± 0.052	5.29 ± 0.450	14.71 ± 0.491	4.53 ± 0.164	23.04 ± 0.321 ^b^	1.72 ± 0.189
30 ºC										
One M/d	21.80 ± 0.32	53.30 ± 2.96 ^b^	59.32 ± 4.060 ^ab^	31.50 ± 2.97	1.14 ± 0.069	5.25 ± 0.493	14.33 ± 0.447	4.32 ± 0.175	22.74 ± 0.641 ^b^	1.86 ± 0.164
Two M/d	22.00 ± 0.41	62.94 ± 3.24 ^a^	59.80 ± 2.870 ^ab^	40.94 ± 3.42	1.06 ± 0.066	6.11 ± 0.749	15.23 ± 0.606	4.69 ± 0.295	24.71 ± 0.993 ^a^	1.88 ± 0.158
*P* values										
Temperature	0.253	0.024	0.542	0.235	0.306	0.607	0.927	0.423	0.083	0.683
Number of meals	0.631	0.914	0.812	0.406	0.515	0.959	0.980	0.342	0.680	0.715
Interaction*	0.126	0.003	0.043	0.092	0.103	0.166	0.102	0.632	0.003	0.809
Partial Eta squared (η²)**	0.027	0.278	0.999	0.174	0.164	0.104	0.085	0.064	0.256	0.025

IBW, initial body weight; FBW, final body weight; M/d, meals/day; TFI, TWG, total weight gain; total feed intake; FCR, feed conversion ratio; SGR, specific growth rate; FL, final body length; FW, final body width; FT, final body thickness; CF, condition factor. *donates the interaction between the water temperature and number of meals per day. **donates the partial Eta Squared (η²) for the temperature-number of meals interaction. The raw data were obtained from Hamed et al. [21]. Different letters indicate statistical significances at *P* < 0.05

### Serum biochemical profiling

Serum glucose reached its lowest significant level in the group raised under 28 ºC and receiving two meals per day. Conversely, the highest significant levels were recorded in the group raised under 26 ºC and 28 ºC while receiving only one meal per day (Table 4). Total protein levels in the serum were significantly higher in the group raised under 28 ºC compared to the other groups. The same trend was observed in globulin levels, with the exception of a similar high level found in fish raised under 30 ºC and receiving one meal per day. Albumin levels did not significantly change among groups, except for lower values observed in fish raised under 30 ºC and receiving one meal per day.

**Table 4:** Serum biochemical profile of the different groups raised under varied temperatures and feeding frequencies

	Glucose (mg/dL)	Total protein (g/dL)	Globulin (g/dL)	Albumin (g/dL)	AST (U/L)	ALT (U/L)
26 ºC						
One M/d	14.82 ± 0.29 ^a^	3.34 ± 0.014 ^b^	1.17 ± 0.017 ^b^	2.17 ± 0.003 ^a^	85.45 ± 3.40 ^a^	6.62 ± 0.072 ^c^
Two M/d	13.81 ± 0.14 ^ab^	3.37 ± 0.049 ^b^	1.23 ± 0.032 ^b^	2.14 ± 0.017 ^a^	76.83 ± 1.95 ^c^	7.55 ± 0.038 ^a^
28 ºC						
One M/d	14.07 ± 0.60 ^a^	3.48 ± 0.009 ^a^	1.41 ± 0.014 ^a^	2.07 ± 0.023 ^a^	82.17 ± 1.22 ^ab^	7.31 ± 0.014 ^b^
Two M/d	11.02 ± 0.25 ^c^	3.54 ± 0.014 ^a^	1.39 ± 0.081^a^	2.15 ± 0.095 ^a^	80.72 ± 0.22 ^b^	7.63 ± 0.098 ^a^
30 ºC						
One M/d	12.80 ± 0.14 ^b^	3.26 ± 0.046 ^b^	1.42 ± 0.14 ^a^	1.85 ± 0.095 ^b^	80.35 ± 0.113 ^b^	7.34 ± 0.023 ^b^
Two M/d	13.02 ± 0.03 ^b^	3.30 ± 0.017 ^b^	1.23 ± 0.026 ^b^	2.08 ± 0.075 ^a^	80.18 ± 0.104 ^b^	7.19 ± 0.040 ^b^
*P* values						
Temperature	< 0.001	< 0.001	0.040	0.017	0.771	< 0.001
Number of meals	< 0.001	0.100	0.394	0.076	0.029	< 0.001
Interaction*	0.001	0.878	0.230	0.135	0.057	< 0.001
Partial Eta squared (η²)**	0.71	0.021	0.217	0.283	0.38	0.887

AST, aspartate aminotransferase; ALT, alanine aminotransferase; M/d, meals/day. *donates the interaction between the water temperature and number of meals per day. **donates the partial Eta Squared (η²) for the temperature-number of meals interaction. Different letters indicate statistical significances at *P* < 0.05

The levels of serum aspartate aminotransferase (AST) were generally similar across groups, except for fish raised under 26 ºC and 28 ºC while receiving one meal per day, which exhibited higher values, and fish raised under 26 ºC while receiving two meals per day, which showed lower values (Table 4). Serum alanine aminotransferase (ALT) levels were high in the group raised under 26 ºC and 28 ºC while receiving two meals per day, while they were low in the group raised under 26 ºC and 28 ºC while receiving one meal per day. The interaction of rearing temperatures and feeding frequencies significantly affected serum glucose and ALT levels (Table 4).

Of interest, cholesterol levels increased in all groups that received two meals, regardless of the rearing temperature (Table 5). Although there were no significant differences within groups in terms of serum levels of triglycerides, MDA, IgM, CAT, SOD, and LYZ, the rearing temperature had a notable effect on the levels of triglycerides, IgM, SOD, and LYZ. Furthermore, the number of meals had a significant impact on the levels of cholesterol, triglycerides, MDA, IgM, and SOD (Table 5).

**Table 5:** Serum lipid profile and activities of antioxidant and immune enzymes of the different groups raised under varied temperatures and feeding frequencies

	Cholesterol (mg/dL)	Triglycerides (mg/dL)	MDA (nmol/g)	IgM (µg/mL)	CAT (U/g)	SOD (U/g)	LYZ (µg/ml)
26 ºC							
One M/d	76.41 ± 3.95 ^b^	102.31 ± 0.191	17.08 ± 0.043	5.21 ± 0.038	17.33 ± 0.038	9.83 ± 0.038	9.96 ± 0.055
Two M/d	82.20 ± 1.13 ^a^	111.91 ± 1.35	18.17 ± 0.098	5.13 ± 0.020	17.44 ± 0.147	9.84 ± 0.014	10.32 ± 0.055
28 ºC							
One M/d	72.29 ± 0.546 ^b^	101.68 ± 0.903	18.11 ± 0.061	5.02 ± 0.012	17.63 ± 0.219	9.90 ± 0.38	10.28 ± 0.023
Two M/d	80.4 ± 0.260 ^a^	85.40 ± 1.703	16.41 ± 0.237	5.47 ± 0.061	17.10 ± 0.153	9.99 ± 0.072	10.06 ± 0.115
30 ºC							
One M/d	78.65 ± 1.74 ^b^	90.57 ± 0.395	16.43 ± 0.245	5.12 ± 0.012	17.47 ± 0.055	9.92 ± 0.162	10.99 ± 0.095
Two M/d	82.09 ± 1.12 ^a^	126.17 ± 2.79	18.13 ± 0.043	5.10 ± 0.055	17.26 ± 0.289	10.34 ± 0.032	10.65 ± 0.205
*P* values							
Temperature	0.159	0.021	0.052	0.014	0.988	0.007	< 0.001
Number of meals	0.002	0.036	0.011	0.003	0.068	0.018	0.467
Interaction*	0.427	0.001	< 0.001	< 0.001	0.079	0.05	0.016
Partial Eta squared (η²)**	0.132	0.692	0.925	0.826	0.346	0.394	0.497

MDA, malondialdehyde; IgM, immunoglobulin M; CAT, catalase; SOD, sodium oxide dismutase; LYZ, lysozyme; M/d, meals/day. *donates the interaction between the water temperature and number of meals per day. **donates the partial Eta Squared (η²) for the temperature-number of meals interaction. Different letters indicate statistical significances at *P* < 0.05

Additionally, a significant interaction was observed between the water temperature and the number of meals per day for all these parameters, except for cholesterol and CAT (Table 5).

### Expression of selected genes related to growth, feed intake, lipid metabolism, antioxidants, and innate immunity

The expression of hepatic *ghr1* was approximately similar among groups that received one meal per day; however, it notably increased in the fish raised under 30 ºC and receiving two meals per day (Fig. 1A). Hepatic *igf1* was markedly increased in the fish raised under 30 ºC, with the most notable value observed in fish that received two meals, recording approximately a 3-fold increase compared to those raised under 28 ºC and receiving two meals per day as a control group (Fig. 1B).

Leptin was downregulated in the group raised under 26 ºC and receiving two meals per day (Fig. 1C). In contrast, it was upregulated in the group raised under 30 ºC, particularly in those received one meal per day. Hepatic *cck* was only upregulated in the group raised under 26 ºC and receiving one meal per day (Fig. 1D). However, it was significantly downregulated in the group raised under 30 ºC and receiving one meal per day.

**Fig. 2:**
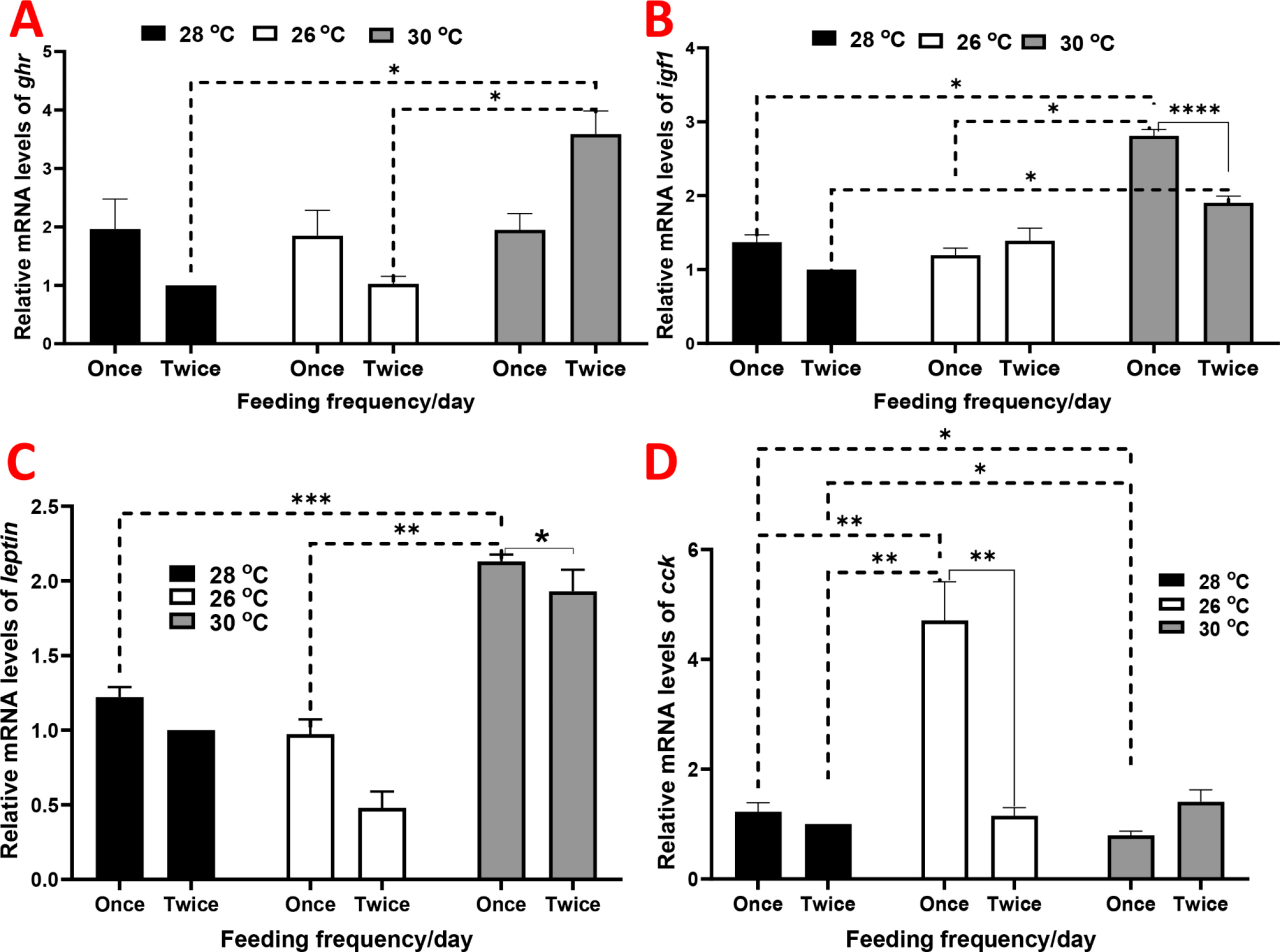
Relative gene expression results of (**A**) growth hormone receptor (*ghr*), (**B**) insulin-like growth factor 1 (*igf1*), (**C**) *leptin*, and (**D**) cholecystokinin (*cck*) in juvenile Nile tilapia (*Oreochromis niloticus*) raised under three distinct water temperatures (26, 28, and 30 ºC) and two feeding frequencies (1 and 2 meals/day) for each temperature over a two-month period. The fish cultured at a water temperature of 28 ºC and fed twice daily served as the control. Data are expressed as means ± SEM. *indicates *P <* 0.05, **indicates *P <* 0.01, and ***indicates *P <* 0.001

The expression of hepatic *fabp* was significantly downregulated in the group raised under 26 ºC, while it was upregulated in the other groups without a significant effect (Fig. 2A). The same trend was observed in the expression of hepatic *cd36*, but with a more significant effect (Fig. 2B).

**Fig. 3:**
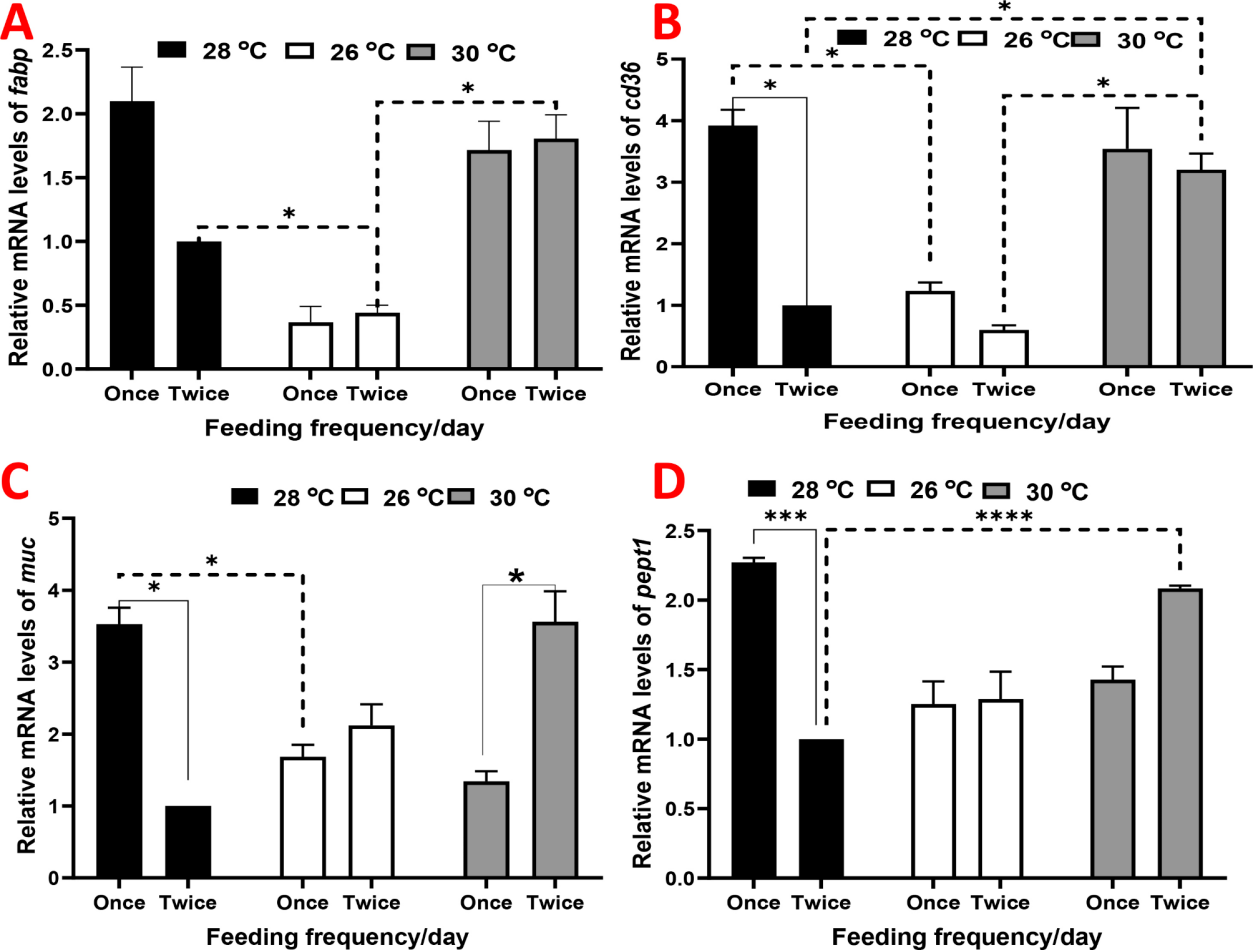
Relative gene expression results of (**A**) fatty acid binding protein (*fabp*), (**B**) intestinal cluster of differentiation 36 (*cd36*), (**C**) mucin-like protein (*muc*), and (**D**) oligo-peptide transporter 1 (*pept1*) in juvenile Nile tilapia (*Oreochromis niloticus*) raised under three distinct water temperatures (26, 28, and 30 ºC) and two feeding frequencies (1 and 2 meals/day) for each temperature over a two-month period. The fish cultured at a water temperature of 28 ºC and fed twice daily served as the control. Data are expressed as means ± SEM. *indicates *P <* 0.05, **indicates *P <* 0.01, and ***indicates *P <* 0.001

The mRNA levels of *muc* and *pept1* exhibited similar increased values in fish raised under 28 ºC and receiving one meal, as well as those raised under 30 ºC and receiving two meals (Fig. 3C and D). The other groups displayed moderate nonsignificant increases when compared to fish raised under 28 ºC and receiving two meals.

**Fig. 4:**
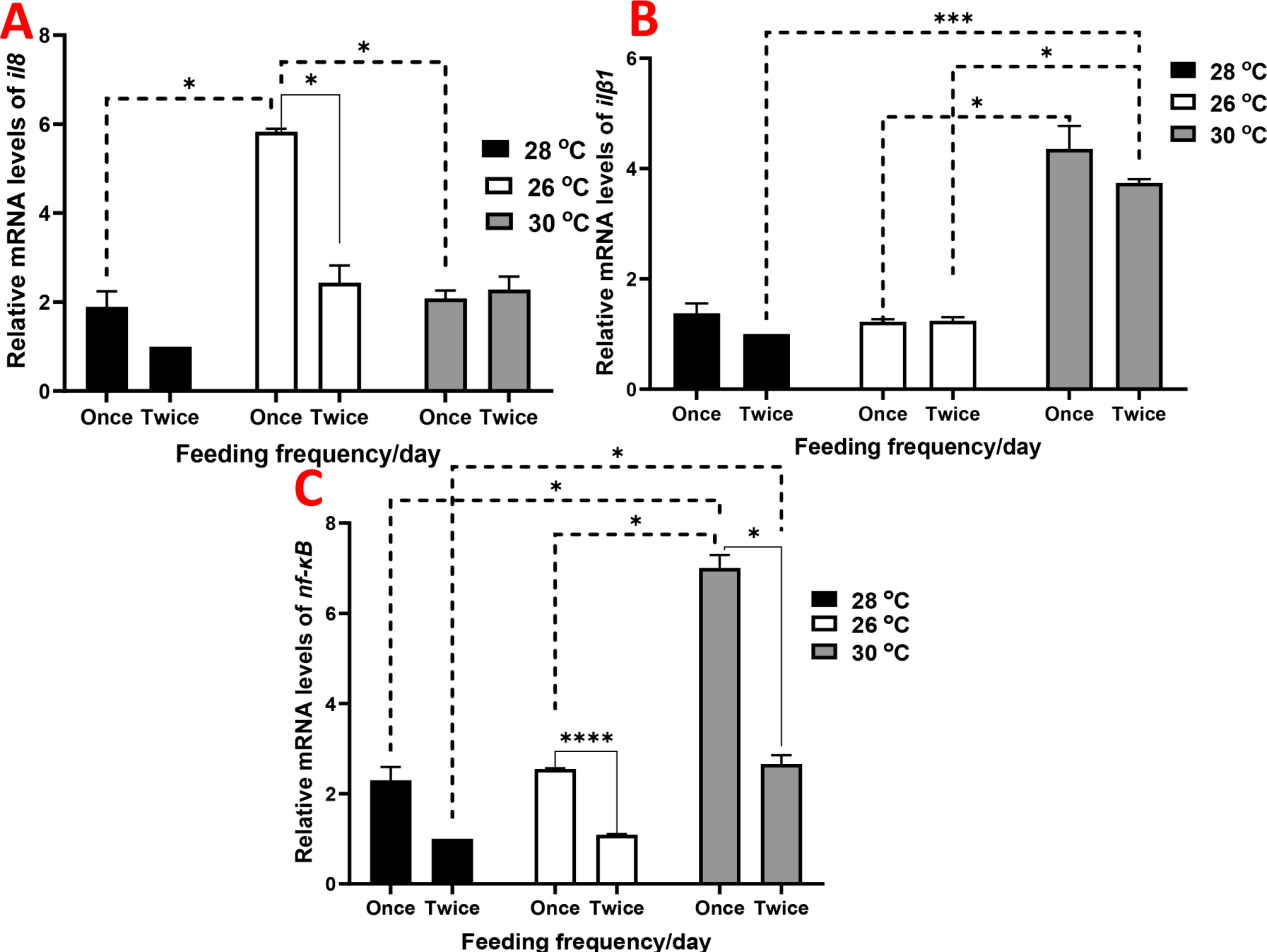
Relative gene expression results of (**A**) interleukin 8 (*il8*), (**B**) interleukin β 1 (*ilβ1*), and (**C**) nuclear factor kappa B subunit (*nf-κB*) in juvenile Nile tilapia (*Oreochromis niloticus*) raised under three distinct water temperatures (26, 28, and 30 ºC) and two feeding frequencies (1 and 2 meals/day) for each temperature over a two-month period. The fish cultured at a water temperature of 28 ºC and fed twice daily served as the control. Data are expressed as means ± SEM. *indicates *P <* 0.05, **indicates *P <* 0.01, and ***indicates *P <* 0.001

**Fig. 5:**
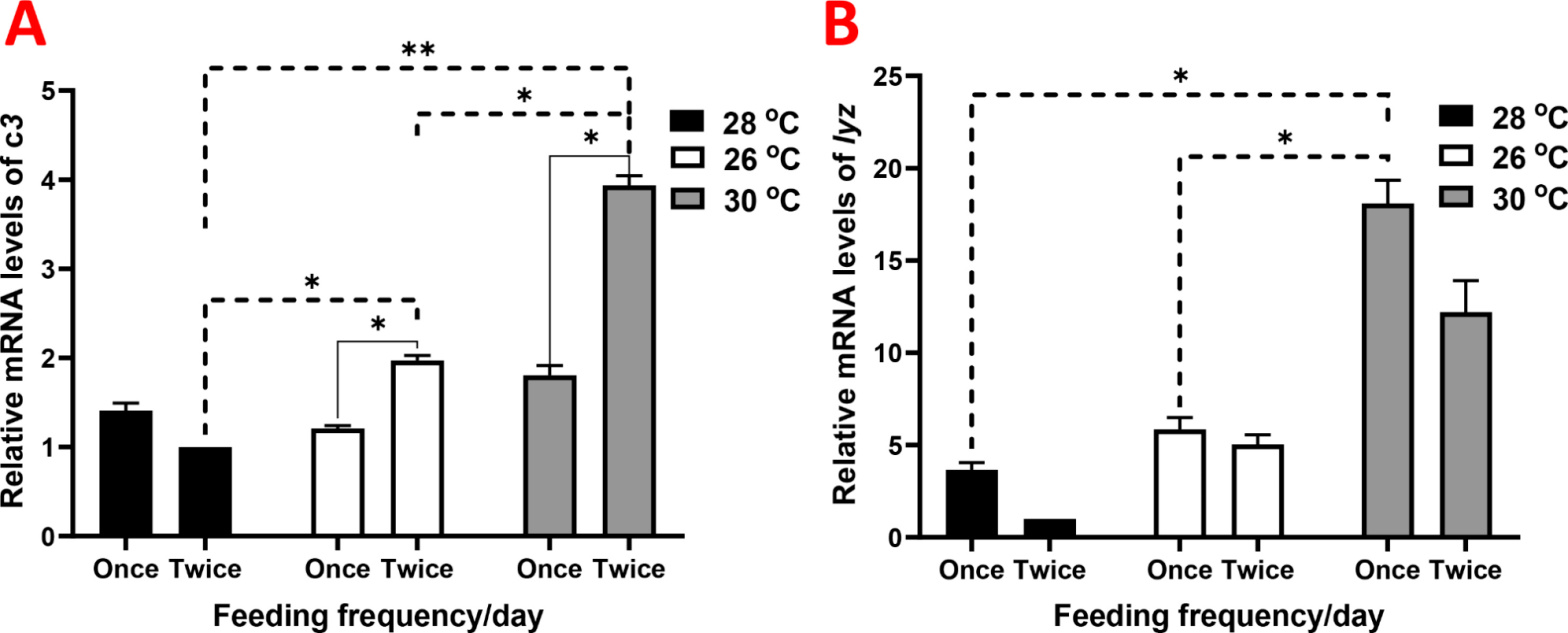
Relative gene expression results of (**A**) complement C3 (*c3*), and (**B**) lysozyme (*lyz*) in juvenile Nile tilapia (*Oreochromis niloticus*) raised under three distinct water temperatures (26, 28, and 30 ºC) and two feeding frequencies (1 and 2 meals/day) for each temperature over a two-month period. The fish cultured at a water temperature of 28 ºC and fed twice daily served as the control. Data are expressed as means ± SEM. *indicates *P <* 0.05, **indicates *P <* 0.01, and ***indicates *P <* 0.001

The expression of hepatic *il8* increased in all groups compared to those raised under 28 ºC and receiving two meals per day, with marked values observed in the group raised under 26 ºC and receiving one meal per day (Fig. 4A). Hepatic *ilβ1* exhibited upregulation in groups raised under 30 ºC, showing more than a 4-fold increase (Fig. 4B). Similarly, *nf-κB* showed more pronounced upregulation, particularly in the group raised under 30 ºC and receiving one meal per day (Fig. 4C). The expression of hepatic *c3* and *lyz* showed gradual increases among groups, reaching notable values in the group raised under 30 ºC and receiving two meals per day and one meal per day, respectively (Fig. 5).

**Fig. 6:**
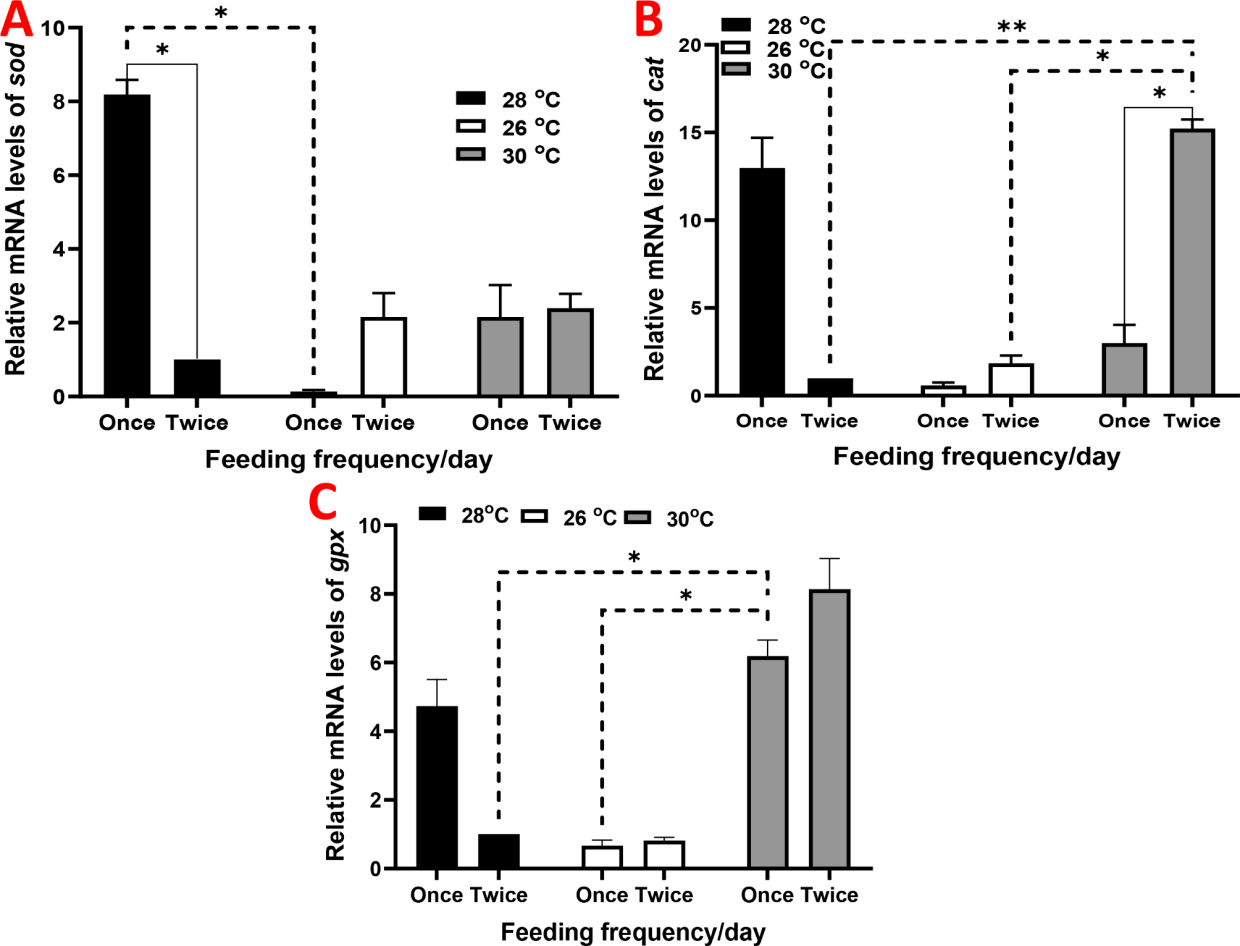
Relative gene expression results of (**A**) superoxide dismutase (*sod*), (**B**) catalase (*cat*), and (**C**) glutathione peroxidase (*gpx*) in juvenile Nile tilapia (*Oreochromis niloticus*) raised under three distinct water temperatures (26, 28, and 30 ºC) and two feeding frequencies (1 and 2 meals/day) for each temperature over a two-month period. The fish cultured at a water temperature of 28 ºC and fed twice daily served as the control. Data are expressed as means ± SEM. *indicates *P <* 0.05, **indicates *P <* 0.01, and ***indicates *P <* 0.001

Compared to the fish raised under 28 ºC and receiving two meals per day, the expression of hepatic *sod*, *cat*, and *gpx* was nonsignificantly increased in groups raised under 28 ºC and receiving one meal per day, except for *sod*, where a significant increase was observed (Fig. 6). In the case of those raised under 30 ºC, the expression of the antioxidant genes was upregulated, with significant values of *cat* levels in the fish raised under 30 ºC and receiving two meals per day, and *gpx* levels in the fish raised under 30 ºC and receiving one meal per day.

## Discussion

The water temperature of the group raised under 30 ºC and 28 ºC recorded increases in water’s electrical conductivity and TDS. It has been found that when water temperature increases, water characteristics change, including viscosity and stratification, resulting in an increase in the water’s charge-carrying capacity [28, 29]. Additionally, increasing water temperature modulates the solubility of ions, leading to increased dissolved salts. These are capable of raising water’s electrical conductivity and TDS [28].

Furthermore, ammonia nitrogen is composed of ammonium and un-ionized ammonia, with their ratio depending on the pH. A pH above 9.75 favors the ratio towards ammonium, while a pH below 8.75 favors the ratio towards un-ionized ammonia [30]. In an estimation, each additional pH unit was found to increase the un-ionized ammonia to ammonium ratio by 10-fold, and each 10 °C rise in temperature in the range 0 to 30 °C increased this ratio by 2-fold [31]. Thus, in the current study, rising temperatures did not greatly affect TAN; additionally, the pH remained approximately constant, resulting in minor changes in TAN. However, a slightly increased TAN may result from increased nitrogenous matter in the water, either from fecal wastes or small uneaten food [32]. Having said that, the TAN was lower than 0.5 mg/L (the safe limit for aquatic organisms’ health) [33].

Water quality, temperature, and their interaction are integral factors influencing the health and growth of aquatic organisms [6, 21, 34, 35]. Notably, the TFI reached its highest significant value in the group raised under 28 ºC, receiving one meal per day, compared to the other groups. However, this trend was not observed in the group raised under 30 ºC, where TFI was nonsignificant. In contrast, FBW and FT showed their highest significance in the group raised under 30 ºC with two meals per day. Moreover, the interaction between temperature and FF proved to be significant concerning FBW, TFI, and FT. This observation extended to the expression of hepatic *ghr1* and *igf1* in the group raised under 30 ºC. Consistently, research has demonstrated that the activation of the GH-IGF1 axis is linked to improved growth performance in Malabar grouper juveniles (*Epinephelus malabaricus*) under isosmotic conditions, without affecting appetite [36]. The activation of the growth hormone receptor via specific modulators such as GH stimulates the Janus kinase 2 (JAK2) and signal transducer and activator of transcription 5B (STAT5) pathway, inducing the synthesis of IGF1 [37]. Additionally, GH has a direct impact on growth by increasing the phosphorylation of JAK2/STAT5, even in models with disrupted *Igfr1* disrupted [38]. The GH-GHR-IGF1 axis plays a critical role in growth and skeletal development, with IGF1 promoting mitochondrial biogenesis and regulating mitochondrial function to enhance metabolism [37, 39]. The response of the Gh/Igf1 axis to food intake has been observed as a reliable indicator of growth dynamics [40]. For instance, plasma Igf1 levels increased upon refeeding food-deprived juvenile gopher rockfish (*Sebastes carnatus*), with *igf1* mRNA levels in the liver also rising after two days of refeeding [40]. This elucidates the significant involvement of the Gh/Igf1 axis in growth and development, emphasizing its crucial role in maintaining an optimal growth rate.

Metabolic activities in fish are triggered by exposure to high temperatures at physiological levels, and this effect persists even after returning to normal rearing temperatures [41]. This metabolic activation results in improved growth, feed efficiency, and protein retention [41]. One key metabolic process affected is lipid metabolism. Notably, the expression of hepatic *fabp* and *cd36* increased in the group raised under 28 °C and received one meal per day, as well as in the group raised under 30 °C, regardless of meal frequency. CD36 and FABP play various roles in lipid metabolism, particularly in skeletal muscles and the heart [42–44]. Synergistically, FABP and CD36 facilitate fatty acid transport and increase protein content in muscle tissue [45]. Rising water temperatures significantly influence metabolomics, particularly lipid metabolism [46]. Water temperature plays a crucial role in regulating fat deposition and conversion in darkbarbel catfish (*Pelteobagrus vachellii*) [47] and even affects the fatty acid composition of tissues [48, 49]. Additionally, FF has a notable impact on lipid metabolism in *Acipenser dabryanus* [50], which higher FF leading to increased lipid and protein accumulation, as well as larger muscle fiber diameter in fish [8, 51].

The temperature and FF also modulate the expression of hormones that regulate growth and feed intake, such as leptin, which plays a crucial role in energy balance and body weight regulation [52]. Hepatic expression of leptin and brain leptin receptor was found to increase during enhanced fish growth and lipid deposition. Their expression was linked to temperature and feeding availability [52, 53]. In contrast, the feeding inhibitory gene (which has an anorexigenic effect), *cck*, is correlated with low FI [53], and its suppression increases FI [54]. Cck is highly responsive to changes in water temperatures [53]. Typically, fish raised under 30 ºC exhibited upregulated leptin and downregulated *cck*, while those raised under 26 ºC showed downregulated leptin and upregulated *cck*, particularly those receiving one meal per day. This suggests that a water temperature of 30 ºC modulates energy homeostasis and metabolic pathways and FI, enhancing growth performance in juvenile Nile tilapia.

Furthermore, leptin suppresses glucagon and corticosterone production, increases glucose uptake, and inhibits hepatic glucose release [55]. Both leptin and cck contribute to regulating blood glucose and cholesterol levels [56–58]. This may explain the variations in serum glucose, cholesterol, and triglyceride levels across the groups, which could be attributed to changes in the expression of these genes. Additionally, CD36 may facilitate cholesterol absorption in the gut [59]. In other species, disruption or knockout of hepatic CD36 reduces triglyceride and cholesterol ester content [60]. Similarly, forced expression of hepatic CD36 has been linked to significant increases in hepatic fatty acid uptake in vivo, contributing to dyslipidemia [61].

In contrast, the expression of hepatic cd36 was higher in fish that received one meal per day, while cholesterol and triglyceride levels were elevated in fish fed two meals per day. This difference could be attributed to species-specific responses or the possibility that one meal per day is insufficient to maintain normal lipid metabolism and liver function. Therefore, a regimen of two meals per day may be more appropriate.

Consistent with this concept, achieving a normal physiological and health state is a crucial milestone in growth performance. A robust connection exists between growth and immune and antioxidant regulators [62, 63]. IGF1 restores normal health and alleviates stress conditions by improving mitochondrial function, including the balancing of metabolism, providing mitochondrial protection, and exhibiting hepatoprotective effects [37]. The GH–GHR–IGF1 axis contributes to balancing mitochondrial mass, cell survival, adaptation, and mitochondrial homeostasis [37, 64].

Interestingly, GH and IGF1 have been shown to increase both the mRNA expression and protein phosphorylation of NF-κB p65, influencing immune-related pathways [38, 65]. This suggests a potential link between growth and immune responses, highlighting the interaction between growth-related genes and their effects on the immune system. Consistent with these findings, rearing temperature significantly affected serum levels of IgM, SOD, and LYZ, in line with the expression of *igf1* and *gh*. Additionally, the number of daily meals had a notable impact on MDA, IgM, and SOD levels. A rearing temperature of 30 °C enhanced the hepatic expression of key immune-related genes such as *muc*, *pept1*, *ilβ1*, *nf-κB*, *c3*, *lyz*, *sod*, *cat*, and *gpx*, with more pronounced effects observed when fish were fed twice daily. However, a temperature of 28 °C with one meal per day also upregulated some of these genes, including *muc*, *pept1*, and *sod*. These genes are major regulatory effectors that modulate immune function and the ability to combat stress and pathogens [66, 67]. Our results indicate that a temperature of 28 °C, combined with feeding twice per day, may provide a more balanced immune response.

The rearing temperature plays a significant role in modulating the immunity of aquatic organisms. In aquatic invertebrates, lower rearing temperatures were found to be beneficial when they were challenged with viral infections, leading to a higher expression of immune genes such as β-1,3-glucan binding protein compared to exposure to lower temperatures [68]. In fish vertebrates, both long- and short-term exposure to suboptimal temperatures can disrupt the immune defense and response against pathogens [69]. Our study indicated that higher temperatures were acceptable and correlated with higher and moderate expressions of immune and antioxidant genes. Similarly, a temperature of 21 ºC demonstrated an optimum immune response in Japanese flounder (*Paralichthys olivaceus*) challenged with viral infection. However, this response was comparable to that of fish exposed to 26 ºC [70]. Consistent with these findings, our study showed increased serum globulin levels in fish raised under 30 ºC and receiving one meal. However, this increase was not significantly different from those raised under 28 ºC. Likewise, parasitic vaccination demonstrated increased antibodies in channel catfish (*Ictalurus punctatus*) exposed to higher temperatures. This effect persisted for up to 20 days post-vaccination [71].

Increases in FF led to elevated levels of ALT and AST in fish fed 3–6 meals per day [72]. In common carp (*Cyprinus carpio*), levels of ALT, AST, and other parameters suggest that FF plays a crucial role in mitigating high-temperature stress [73]. This indicates an important interaction between FF and rearing temperature, which is essential for maintaining proper bodily functions. In the current study, based on hepatic health indicators (ALT and AST), ALT and AST levels did not change significantly in response to rising temperatures, with only slight variations in response to changes in FF. This suggests good liver health and reflects a normal physiological state.

The same principle also applies to FF, where serum LYZ activity and total hemolytic complement were lower in fish fed 1–2 times per day compared to those fed 3–6 times per day. However, the defense against hypoxia stress remained in good condition in juvenile dolly varden char *Salvelinus malma* when receiving one or two meals per day [72]. In juvenile blunt snout bream *Megalobrama amblycephala*, both low (2 meals per day) and high FF (3 meals or more per day) resulted in decreased liver CAT and GPX activities, as well as nonspecific immune parameters such as LYZ, total hemolytic complement, acid phosphatase, myeloperoxidase, and nitric oxide [19]. Garcia and Villarroel [74] concluded that increasing FF could enhance immune response and improve disease resistance in Nile tilapia. This suggests that determining the optimum FF and rearing temperature is critical in aquaculture to achieve the highest immune response and health status.

Rearing temperature and FF are key factors that regulate fish performance. Future research could explore glucose-related and other signaling pathways, as well as include additional organs such as the gut, spleen, and kidney, to further deepen our understanding of how these metabolic processes are influenced by environmental conditions.

## Conclusion

The results indicate that water temperature and feeding frequencies play a crucial role in shaping body growth performance and immune response. While rearing at a water temperature of 28 ºC with a feeding frequency of two meals per day maintained good body physiology, increasing the temperature to 30 ºC, particularly with two meals per day, also proved beneficial, enhancing the overall health and growth performance of juvenile Nile tilapia. Conducting further studies that incorporate additional factors, such as pathogen challenges, would provide more insights into the effects of a temperature of 30 ºC.

## Data Availability

Data & materials are available upon reasonable request from the co-corresponding author.
